# A Case of Pyelonephritis in an Anuric Patient with End-Stage Renal Disease on Hemodialysis

**DOI:** 10.7759/cureus.15353

**Published:** 2021-05-31

**Authors:** Sherif Elkattawy, Islam Younes, Hardik Fichadiya, Abraheim Al-nasseri, Aravinda Reddy

**Affiliations:** 1 Internal Medicine, Rutgers New Jersey Medical School/Trinitas Regional Medical Center, Elizabeth, USA; 2 Internal Medicine, Trinitas Regional Medical Center, Elizabeth, USA; 3 Internal Medicine, St. George's University School of Medicine, Elizabeth, USA; 4 Nephrology, Trinitas Regional Medical Center, Elizabeth, USA

**Keywords:** pyelonephritis, end-stage renal disease (esrd), kidney, hemodialysis, urinary tract infection

## Abstract

Urinary tract infections are common sources of infections requiring antibiotic use worldwide. Chronic kidney disease (CKD) patients, especially those with minimal urine output are challenging when it comes to antibiotic stewardship given the scarcity of cases in the literature. It is further complicated by the fact that end-stage renal disease (ESRD) patients are found to have asymptomatic pyuria and its clinical significance for bacterial infection is yet to be determined. In this case, we report a patient who presented with non-specific symptoms including fever, cough, chills, diarrhea, nausea and was found to have left-sided perinephric stranding on CT scan of the abdomen. The patient also had a fever of 104.6 F which we attributed to left-sided pyelonephritis given the high sensitivity of such findings on CT scan. In this report, we explored the literature for the incidence and management of pyelonephritis in ESRD patients who are anuric.

## Introduction

Urinary tract infections are one of the non-access-related infections in chronic dialysis patients. Patients with residual urine production usually present with the same clinical picture as patients without kidney disease. However, anuric patients may present with only bladder discomfort. Asymptomatic pyuria without bacterial infection is common in chronic dialysis patients, however, its clinical significance for bacterial infection is controversial. Most of the studies have shown no correlation between the presence of white blood cells and urinary tract infection in asymptomatic dialysis patients [1-2]. Moreover, urinary tract infection is the most common nosocomial infection in dialysis patients who have undergone urinary catheterization [3]. Patients with end-stage renal disease (ESRD) due to polycystic kidney disease have an increased risk of pyelonephritis. We present a case of a young female with ESRD who presented with high-grade fever and was found to have left renal pyelonephritis.

## Case presentation

A 24-year-old female with a past medical history of systemic lupus erythematosus (SLE), ESRD on hemodialysis, chronic anemia, secondary hyperparathyroidism, hypertension, systolic heart failure, anxiety disorder, and panic disorder presented to the emergency department complaining of resting, non-positional chest pain, nausea, non-bloody/non-bilious vomiting, productive cough, diarrhea, subjective fever and chills of one-day duration. Of note, she was discharged from the hospital one day prior for hypertensive emergency with flash pulmonary edema secondary to non compliance with hemodialysis sessions. A triple lumen central venous catheter was introduced into the left internal jugular vein during that admission which was later removed prior to her discharge from the hospital.

On presentation, her rectal temperature was 104.6 Fahrenheit, heart rate was 102 beats/min, respiratory rate was 29 breaths/min, blood pressure was 148/89 mm Hg, and oxygen saturation was 96% on room air. Physical exam was significant for rales in bilateral upper lung fields and epigastric tenderness, but with no guarding or rigidity. Open pus draining cutaneous abscess was noted on the left neck at the site where the catheter was introduced during the previous admission. Code sepsis was called in the emergency department and the patient was started on intravenous hydration. Serum lactic acid, blood cultures, stool cultures, stool Clostridium difficile antigen tests, and cultures from the neck abscess were sent.

**Figure 1:**
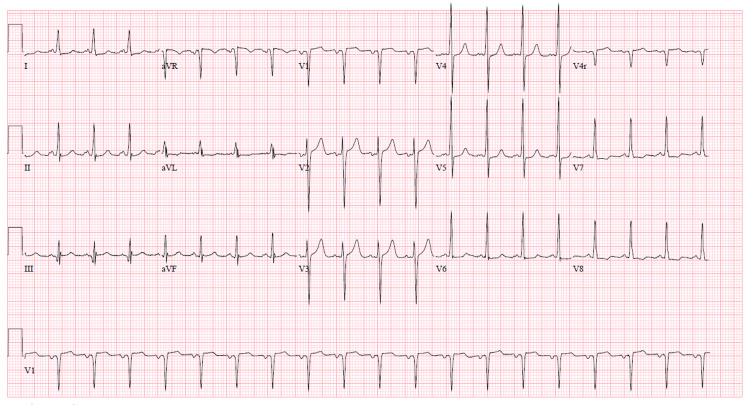
EKG showing left ventricular hypertrophy with no ST-T wave changes suggestive of ischemia

**Figure 2:**
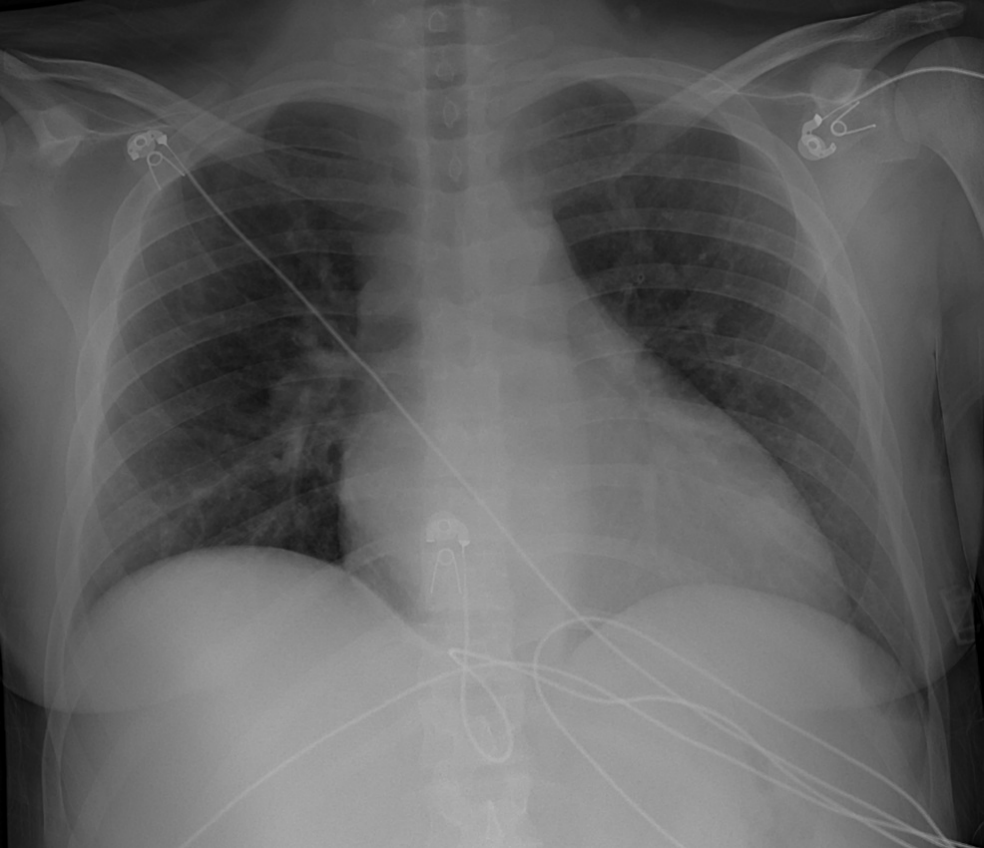
Chest X-ray showed clear lung fields with no signs of consolidation

**Figure 3:**
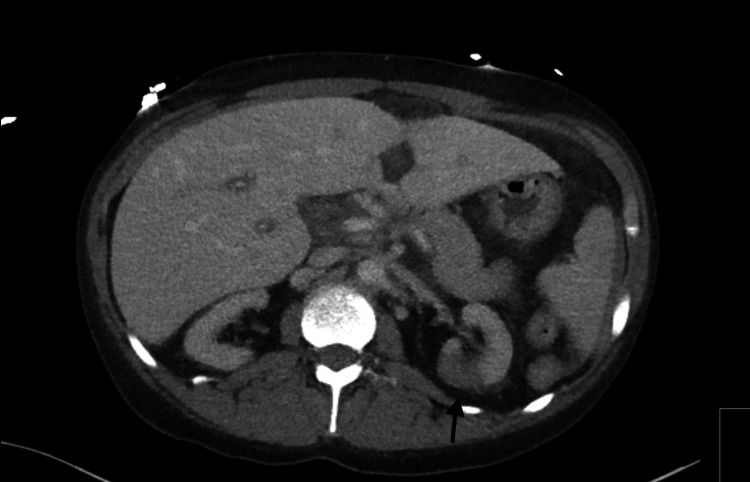
CT abdomen significant for left-sided perinephric stranding as shown by the arrow

Labs were significant for blood urea nitrogen (BUN): 76 mg/dl (8-20), serum creatinine: 10.44 mg/dl (0.4-1.0), serum sodium: 128 MMOL/L (136-144), serum chloride: 89 MMOL/L (101-111), serum bicarbonate: 18 MMOL/L (22-32), anion gap: 21 MMOL/L (3-9), hemoglobin: 10.9 gm/dl (12-16, patients baseline <10), white blood cell count: 14,600/UL (4.8-10.8) with 93% polymorphonuclear cells (42%-75%), platelet count decreased from 147,000 U/L to 56,000U/L over the course of two days (130,000-400,000 U/L), d-dimer: >5000 ng/ml (0-230 ng/ml), fibrinogen: 545 mg/dl (270-500), and serum troponin: 0.18 ng/ml (<0.5). Electrocardiogram (ECG) showed sinus tachycardia with left ventricular hypertrophy but no acute ST or T wave abnormalities as seen in Figure 1. Brain natriuretic peptide: 1354 pg/ml (<100 pg/ml) from 5000 ng/ml six days prior to presentation. COVID 19 polymerase chain reaction (PCR) test was negative. Urine drug screen was positive for marijuana. Chest X-ray showed clear lung fields with no signs of consolidation as seen in Figure 2. CT of the abdomen was suspicious for left kidney pyelonephritis with focal hypo-density at the mid and lower pole associated with minimal peri-nephric fat stranding as seen in Figure 3. 

**Figure 4:**
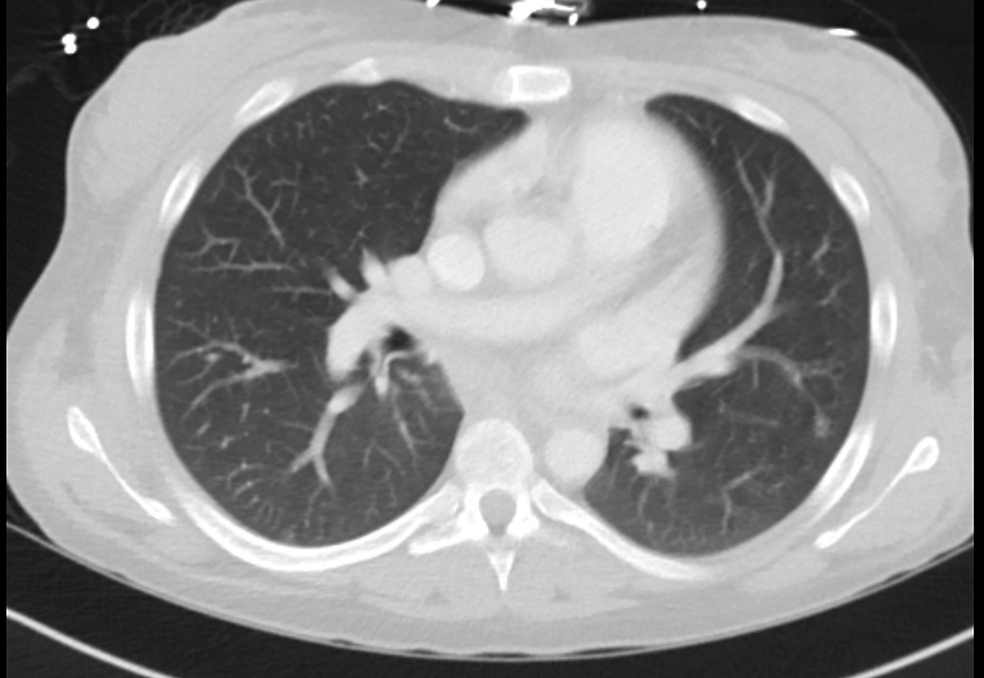
CT chest with contrast was unremarkable for pulmonary embolism

The patient was started on broad-spectrum antibiotics in the emergency department accounting for her history of penicillin allergy. Soon after admission, she became hypotensive and was started on norepinephrine drip along with stress dose of corticosteroids (40 mg IV push followed by 100 mg every eight hours). Infectious disease and surgery were consulted. The neck abscess was drained, however, the surgical team had reported that it was superficial and an unlikely cause of systemic symptoms. Repeat complete cell count showed a platelet count of 31,000/UL (dropped from 56,000/UL) when tested in sodium citrate (blue topped) bottle. Hematology-Oncology was consulted who attributed the thrombocytopenia to sepsis at this point. Anticoagulation was held in the light of worsening thrombocytopenia. Lower extremity venous duplex was negative for deep vein thrombosis (DVT) in bilateral lower extremities. CT chest with contrast was unremarkable for pulmonary embolism as seen in Figure 4. 

Troponin levels continued to trend upward during hospital stay (0.18 ng/ml > 1.78 ng/ml > 5.39 ng/ml > 6.66 ng/ml) and cardiology was consulted. The patient was not complaining of any chest pain at that time. Findings were thought to be secondary to demand ischemia vs myocarditis from lupus flare. An echocardiogram (ECHO) showed left ventricular ejection fraction of 35%-40% with moderately dilated left atrium and moderate tricuspid regurgitation, unchanged from prior echocardiograms. Carvedilol and angiotensin-converting enzyme inhibitors were held in the light of hypotension. The patient was not a candidate for anti-coagulation as outlined above. 

Complement levels were C4: 8 (15-57), C3: 48 (83-193) and CH50: <10 (31-60). The following day, the patient became afebrile, blood pressure stabilized and no pressure support was required, however, her platelet counts continued to drop to a nadir of 36,000/UL. Blood cultures and neck abscess cultures grew Staphylococcus aureus sensitive to vancomycin and resistant to penicillin G. Next day in the afternoon, the patient had a brief episode of mild chest tightness during which ECG showed ST depression in lateral leads (V5, V6, I and aVL) and lead II. Left atrial enlargement and left axis deviation were also noted. In the evening, she had sudden bradycardia and asystole. Telemetry showed broad QRS complexes, followed by sinusoidal waves and asystole. Code blue was called. Advanced cardiac life support (ACLS) protocol was followed. Resuscitation efforts were done for 1 hour and 15 minutes. She was given multiple doses of calcium chloride, magnesium sulphate, and sodium bicarbonate all of which failed to be of any benefit. Bedside ECHO showed very poorly contracting right ventricle but no evidence of right ventricular dilation or strain. The patient was then pronounced dead. 

## Discussion

ESRD or chronic kidney disease (CKD) is an irreversible and debilitating condition that requires prompt treatment with frequent dialysis, hemofiltration, hemodiafiltration, renal replacement therapy, or kidney transplant. More commonly, ESRD is a complication of other chronic disease such as diabetes, SLE, hypertension, polycystic kidney disease, analgesic misuse, or amyloidosis [4]. In addition, CKD commonly results in frequent urinary tract infections secondary to increased inflammation, defective barrier function, or dialysis treatment in general [5-8]. There are more than 7 million urinary tract infections per year reported in the United States, 250,000 of these cases consist of episodes of pyelonephritis [8]. However, it still remains unclear how many of these are attributed to CKD [8]. In spite of the limited research, it still remains unclear how prevalent acute pyelonephritis superimposed by ESRD is documented in patients with CKD, and which antibiotic regimen is the most efficacious.

Our case presents a 24-year-old female with ESRD secondary to lupus nephritis who presented to the emergency department for intractable nausea, vomiting, diarrhea, and coughing; after workup, the patient was diagnosed with a sepsis likely secondary to pyelonephritis as abscess was superficial and unlike source as per surgical evaluation. The patient was started on broad-spectrum antibiotics: vancomycin, and aztreonam after blood/abscess cultures were drawn. Given the high sensitivity of perinephric stranding finding on CT scan for pyelonephritis and septic shock state the patient presented with initially, pyelonephritis was diagnosed based on imaging. We attributed the origin of acute pyelonephritis to a transcending infection of the residual urine in the bladder. It is illustrated in this report that our anuric patient was diagnosed with pyelonephritis in the setting of ESRD and was successfully treated with antibiotics. 

Research attributed to urinary tract infection in patients with CKD is limited, which makes the choice of antibiotic treatment more challenging. Early research from Bennett et al. had concluded that ampicillin and trimethoprim-sulfamethoxazole are sufficient in treating urinary tract infections in anuric patients with renal failure [9]. This finding was later fine-tuned by Gilbert et al. who reported that fluoroquinolones (ciprofloxacin, levofloxacin) and trimethoprim are sufficient in treating urethritis/cystitis in patients with CKD. Furthermore, their research concluded that nitrofurantoin had limited efficacy and should not be used due to low urine drug concentrations [8]. Due to the scarcity of cases, there is limited evidence to support or reject the use of other broad-spectrum antibiotics such as vancomycin or aztreonam in the treatment of pyelonephritis in anuric patients with ESRD.

## Conclusions

Urinary tract infection should be in the differential diagnosis in chronic dialysis patients, especially with residual urine production, presenting with fever. Given the scarcity of cases in the literature, no guidelines exist for antibiotic stewardship of these patients. Early diagnosis and proper antibiotic selection are vital to decrease the mortality in such patients. More research is needed to prove if a superior or inferior treatment regimen for anuric patients with pyelonephritis in the setting of ESRD exists.
